# Effect of Laser Irradiation on Cell Function and Its Implications in Raman Spectroscopy

**DOI:** 10.1128/AEM.02508-17

**Published:** 2018-04-02

**Authors:** Xiaofei Yuan, Yanqing Song, Yizhi Song, Jiabao Xu, Yinhu Wu, Andrew Glidle, Maggie Cusack, Umer Z. Ijaz, Jonathan M. Cooper, Wei E. Huang, Huabing Yin

**Affiliations:** a School of Engineering, University of Glasgow, Glasgow, United Kingdom; b Department of Engineering Science, University of Oxford, Oxford, United Kingdom; c Division of Biological & Environmental Sciences, Faculty of Natural Sciences, University of Stirling, Stirling, United Kingdom; University of Buenos Aires

**Keywords:** single cell, microfluidics, Raman spectroscopy, viability, laser, growth, Raman-activated cell sorting

## Abstract

Lasers are instrumental in advanced bioimaging and Raman spectroscopy. However, they are also well known for their destructive effects on living organisms, leading to concerns about the adverse effects of laser technologies. To implement Raman spectroscopy for cell analysis and manipulation, such as Raman-activated cell sorting, it is crucial to identify nondestructive conditions for living cells. Here, we evaluated quantitatively the effect of 532-nm laser irradiation on bacterial cell fate and growth at the single-cell level. Using a purpose-built microfluidic platform, we were able to quantify the growth characteristics, i.e., specific growth rates and lag times of individual cells, as well as the survival rate of a population in conjunction with Raman spectroscopy. Representative Gram-negative and Gram-positive species show similar trends in response to a laser irradiation dose. Laser irradiation could compromise the physiological function of cells, and the degree of destruction is both dose and strain dependent, ranging from reduced cell growth to a complete loss of cell metabolic activity and finally to physical disintegration. Gram-positive bacterial cells are more susceptible than Gram-negative bacterial strains to irradiation-induced damage. By directly correlating Raman acquisition with single-cell growth characteristics, we provide evidence of nondestructive characteristics of Raman spectroscopy on individual bacterial cells. However, while strong Raman signals can be obtained without causing cell death, the variety of responses from different strains and from individual cells justifies careful evaluation of Raman acquisition conditions if cell viability is critical.

**IMPORTANCE** In Raman spectroscopy, the use of powerful monochromatic light in laser-based systems facilitates the detection of inherently weak signals. This allows environmentally and clinically relevant microorganisms to be measured at the single-cell level. The significance of being able to perform Raman measurement is that, unlike label-based fluorescence techniques, it provides a “fingerprint” that is specific to the identity and state of any (unlabeled) sample. Thus, it has emerged as a powerful method for studying living cells under physiological and environmental conditions. However, the laser's high power also has the potential to kill bacteria, which leads to concerns. The research presented here is a quantitative evaluation that provides a generic platform and methodology to evaluate the effects of laser irradiation on individual bacterial cells. Furthermore, it illustrates this by determining the conditions required to nondestructively measure the spectra of representative bacteria from several different groups.

## INTRODUCTION

As powerful monochromatic light sources, lasers have underpinned the development of modern optical technologies such as optical tweezers, superresolution imaging, and advanced spectroscopic technologies (1), all of which have enabled a rapid expansion of our understanding in the life and environmental sciences. Similarly, the advent of lasers in the 1960s meant that Raman spectroscopy became a practical and affordable technology that has been rapidly implemented in many fields (2–4). In contrast to fluorescence techniques that detect known fluorescent labels, Raman spectroscopy does not require external labeling of samples or any *a priori* knowledge. Instead, it provides a full spectrum of Raman “fingerprints” specific to the intrinsic chemical composition of a sample and thus has emerged as a powerful label-free method for studying living cells directly under their physiological conditions (5). To date, Raman spectroscopy has been widely used for identifying cell phenotypes and functional changes caused by cell processes such as aging and differentiation (3, 6–8). In the last decade, Raman spectroscopy has become increasingly important for studying environmental and clinical microorganisms, the majority of which are not cultivable in the laboratory and are largely unknown (4, 9).

Similar to many other bioimaging and spectroscopic techniques, most lasers used for Raman spectroscopy are in the visible and near-infrared ranges. The interaction between laser light and a molecule produces inelastic scattered light, i.e., light with a different wavelength to that of the incident light, giving rise to a unique Raman shift. Since only approximately 1 in 10^6^ to 10^10^ incident photons generate inelastic scattered light, intrinsic Raman signals are inherently weak (10), and detecting them necessitates the use of high irradiation energy. However, intense laser irradiation can damage biological samples and indeed has been used to kill bacteria for the purpose of disinfection (11, 12). In the reported literature, there are many discrepancies between the observed nondestructive/destructive effects caused by similar conditions applied to bacterial cells. For example, in addition to killing cells, regrowth of bacterial colonies has been found to occur after large doses of laser irradiation (12); in some cases, low-power laser irradiation has also been found to promote bacterial proliferation (13). Surprisingly, studies of laser irradiation on individual bacterial cells have been very limited, despite the well-known fact that there is heterogeneity even in an isogenic population (14).

Here, we exploit single-cell microfluidics for the quantitative evaluation of laser irradiation on bacterial cell growth and fate. The approach enables real-time tracking of the growth of individual cells and thus reveals hidden heterogeneities within a population (14–17). In this study, we used a 532-nm laser as the light source because of its wide use in Raman spectroscopy as well as its potential to damage living cells (11, 18). To investigate differences between species, representative Gram-negative (Escherichia coli JM109 and Acinetobacter baylyi ADP1) and Gram-positive (Bacillus subtilis 168 and Rhodococcus sp. RC291) bacterial strains were investigated. These strains were chosen because they are widely used in microbiology and therefore provide well-characterized systems to investigate the effects of laser irradiation. Considering the requirement to maintain cell function throughout Raman spectroscopic measurements, the growth characteristics of individual cells exposed to a wide range of irradiation conditions were quantified and correlated with their Raman spectra.

## RESULTS

### Real-time monitoring of cell response to laser irradiation.

**FIG 1:**
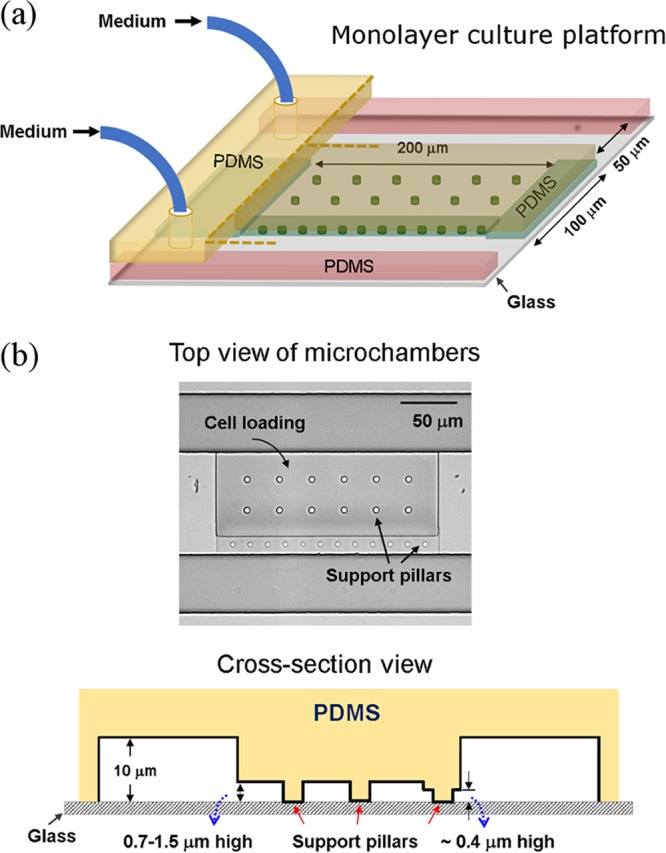
(a) Microfluidic platform used for monolayer cell culture. The microfluidic part of the chip is made of polydimethylsiloxane (PDMS) elastomer, which is bonded to a glass substrate to form an enclosed device. The support pillars in the microchambers prevent potential collapse of the chambers during the bonding process. (b) Top view of a microchamber and cross-section view of the platform.

**FIG 2:**
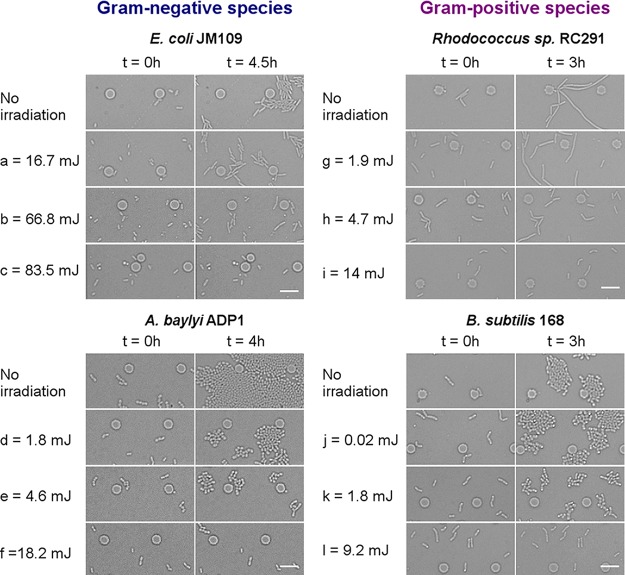
Time-lapse images of monolayer-cultured bacteria immediately after laser irradiation (*t* = 0) and post culture. All the cells in the images (*t* = 0) were exposed to irradiation. The values on the left sides of the images are representative irradiation doses that induced similar changes in each strain. Scale bar, 10 μm.

The monolayer culture of cells within the microfluidic device (Fig. 1) enabled both a focused irradiation of known power and duration to be applied to randomly selected individual cells and their subsequent growth to be tracked. Thus, using a single device, multiple irradiation conditions can be investigated simultaneously on the same chip. This enables a reliable comparative evaluation under the same culture conditions to be made. In this study, each strain was exposed to a series of similar irradiation conditions, from low to high doses. Representative time-lapse images of the four strains from the onset of irradiation (*t* = 0 h) at the selected low, medium, and high doses are shown in Fig. 2. For all the strains, the colonies started initially from single cells, and their growth decreased with increasing irradiation doses (laser power [mW] × acquisition time [s]). No cell division was observed at high doses of laser irradiation (Fig. 2c, f, i, and l); however, the absolute values at which the cells stopped dividing were specific to each strain. This phenomenon suggests that laser irradiation can compromise bacterial growth, and the effect depends on both species and irradiation conditions.

### Effect on cell growth at the single-cell level.

**FIG 3:**
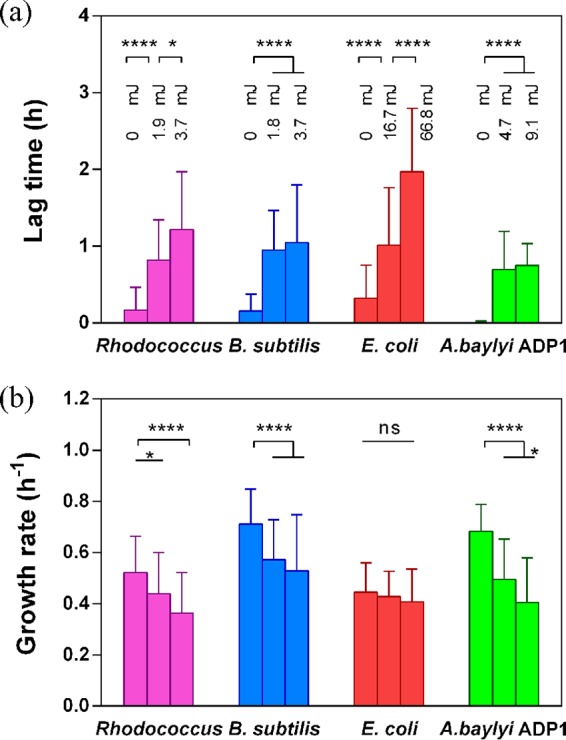
Irradiation-induced variations in the average lag times (a) and growth rates (b) of the bacterial strains under various irradiation doses as indicated in panel a. *, *P* < 0.05; ****, *P* < 0.0001; ns, not significant.

For the four strains tested, it is clear that individual cells of the same strain responded differently to the same irradiation stress. Both continued and staggered growth were observed within a certain range of irradiation doses, clearly demonstrating the survival heterogeneity within a strain as shown previously (16, 17, 19). To understand how the laser irradiation affects cell function, a quantitative evaluation of bacterial growth was carried out at the single-cell level. For the growing cells, two characteristic growth parameters, namely, the specific growth rate (μ) and the lag time (λ), were derived from time-lapse images of singe colonies (16), which provide information of how individual cells respond to the imposed irradiation stress. Below a certain level of irradiation, the average μ value of the dividing cells from the three strains, namely, Rhodococcus sp. RC291, A. baylyi ADP1, and B. subtilis 168, decreased rapidly with increased irradiation dose (*P* < 0.0001) (Fig. 3a). In the case of E. coli JM109, there was no significant decrease in the average μ value even at a very high dose (66.8 mJ). However, all the strains showed increased lag times with increasing doses (*P* < 0.0001) (Fig. 3b), suggesting they delayed their growth to resist the irradiation stress. In particular, the lag time of E. coli JM109 was extended substantially at each elevated dose (*P* < 0.001) (Fig. 3b), which may account for its largely unchanged growth rate.

**FIG 4:**
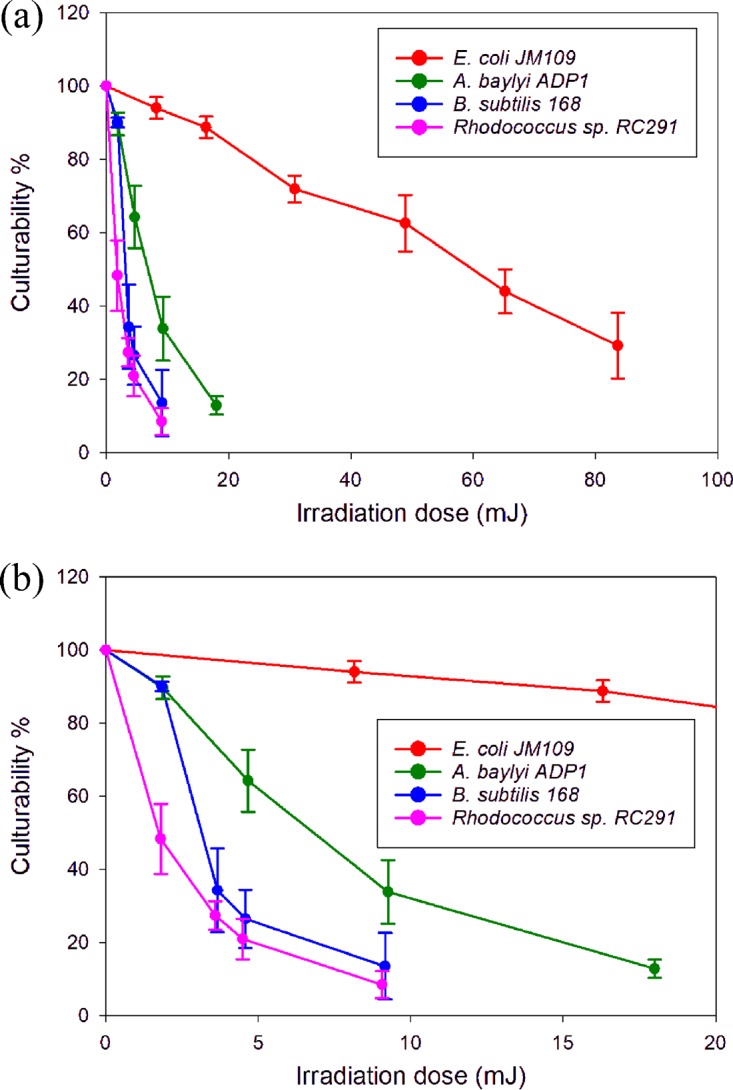
(a) Relationship between culturability of the strains and irradiation doses. (b) Enlarged view of the curves in the range of low irradiation doses (dose < 20 mJ).

For each condition, there were a number of nondividing cells, and thus cell culturability, the percentage of dividing cells in the population of the irradiated cells, was used to illustrate the susceptibility of a strain to laser damage. As shown in Fig. 4, the culturability of the two Gram-positive strains reduced rapidly with the irradiation dose. An irradiation of ∼15 mJ reduced their culturability to close to zero. However, the same dose only reduced that of A. baylyi ADP1 and E. coli to ∼20% and ∼90%, respectively. Together with the relative high growth rates (Fig. 3a) for the dividing cells under these conditions, these results resonate with other evidence for the robustness of the two Gram-negative strains and the difficulty in harming them by irradiation (20).

### Destructive effects on cell viability and integrity.

**FIG 5:**
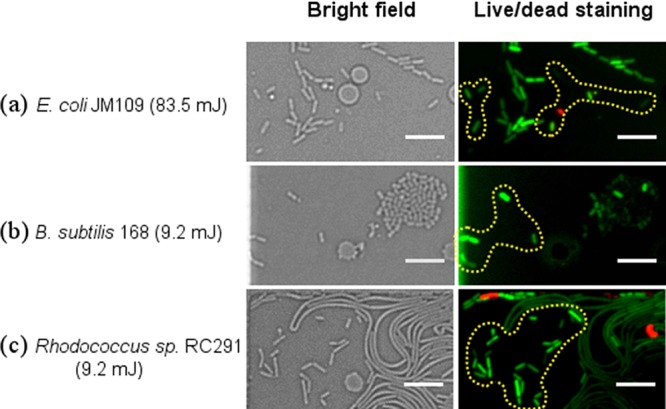
Bright field and live (green)/dead (red) staining images of cells at 6 h in culture after irradiation at doses of 83.5 mJ for E. coli JM109 (a), 9.2 mJ for B. subtilis 168 (b), and 9.2 mJ for Rhodococcus sp. RC291 (c). It should be noted that 10% culturability was observed at these irradiation conditions (see Fig. 4). Only cells denoted in the yellow dotted shapes were illuminated. Scale bar, 10 μm.

Above a certain level of irradiation, some cells stopped dividing (Fig. 2). The dose needed to reach this level, denoted as the “destruction threshold,” was different for each strain and significantly lower for the Gram-positive strains (Table 1). To evaluate whether the nondividing cells were still alive, live/dead staining (SYTO 9/propidium iodide staining; see the supplemental material) was performed at the end of culture periods where the illumination dose resulted in ∼10% of culturability for each strain. SYTO 9 (green) stains nucleic acids of both live and dead cells, whereas propidium iodide (red) only stains dead cells with compromised membranes. As shown in Fig. 5, almost all the nondividing cells of the four strains showed only SYTO 9 fluorescence, indicative of intact cell membranes. However, an intact cell membrane alone is not sufficient evidence of cell viability.

**TABLE 1:** Estimated laser doses versus culturability

Species	Laser dose (mJ) at culturability of:		
	0%	10–50%	50–100%
Gram positive			
Rhodococcus sp. RC291	>12	2–9	<2
B. subtilis 168	>14	2–10	<2
Gram negative			
E. coli JM109	>120	49–84	<49
A. baylyi ADP1	>23	7–19	<7

**FIG 6:**
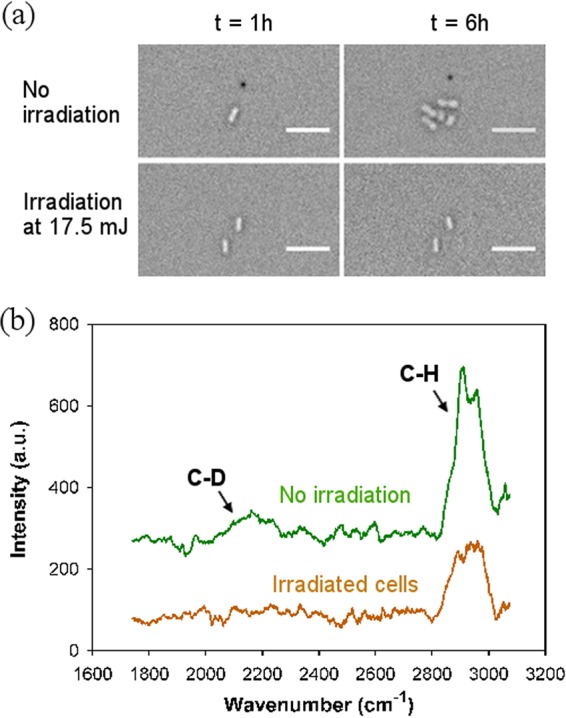
(a) Bright field images of A. baylyi ADP1 with and without irradiation at *t* = 1 h and *t* = 6 h when cultured in the microwell microfluidic device using 90% D_2_O-containing LB. Irradiation conditions for each case are shown on the left side of the images. Scale bar, 10 μm. (b) Single-cell Raman spectra of A. baylyi ADP1 after 6 h culture in D_2_O-containing LB. Each spectrum is an average from 10 cells.

It is acknowledged that viable cells are metabolically active even without obvious growth. Recently, Raman spectroscopy in conjunction with the stable-isotope technique has provided a simple method to evaluate the *in vivo* metabolic activity of a cell, as a consequence of metabolically active bacteria incorporating deuterium from a D_2_O-containing medium into cellular components. This gives rise to a characteristic C-D Raman peak (2,040 to 2,300 cm^−1^) (21). Therefore, this method was employed for the real-time detection of metabolic activity of cells cultured in a two-layered microwell microfluidic device (see Fig. S1). As shown in Fig. 6a, nonirradiated A. baylyi ADP1 cells grew in D_2_O medium in the same manner as in normal H_2_O medium. Their single-cell Raman spectra taken at the end of 6 h in culture show both a C-H peak (2,850 to 3,000 cm^−1^, associated with lipids) and a C-D peak (2,040 to 2,300 cm^−1^) (Fig. 6b). However, Raman spectra of nondividing cells irradiated at 17.5 mJ show only a small C-H peak (2,850 to 3,000 cm^−1^), despite using the same culture and Raman acquisition conditions as for their nonirradiated counterparts. These results showed that these nondividing cells had lost metabolic activity.

**FIG 7:**
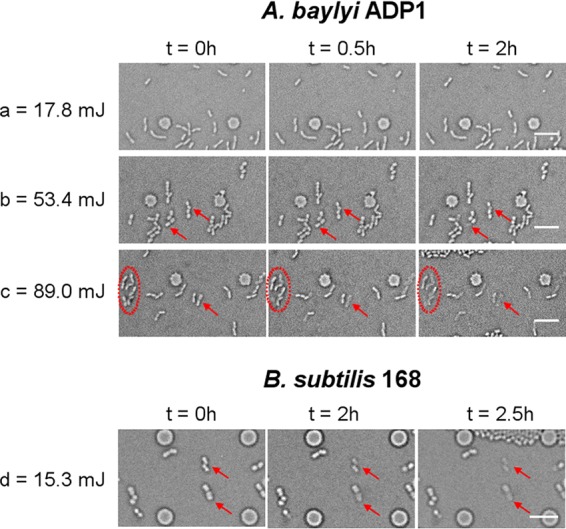
Time-lapse images of monolayer culture of A. baylyi ADP1 (a, b, and c) and B. subtilis 168 (d) after laser irradiation at the indicated doses. Arrows and circles in the images show cells that significantly decomposed with time. Scale bar, 10 μm.

Beyond the “destructive threshold” where cell culturability was reduced to zero, physical cell destruction occurred. For example, when A. baylyi ADP1 cells were under irradiation of 53.4 mJ, i.e., 3 times the 17.5 mJ where nondividing cells was observed, the irradiated cells started to disappear after 2.5 h in culture (Fig. 7a). Furthermore, this dissolution process occurred earlier with an increased dose. For instance, dissolution started 30 min following irradiation of 89.0 mJ (Fig. 7c). A similar phenomenon was observed in the Gram-positive strain, Bacillus subtilis, although the energy needed was much lower (e.g., 5.3 mJ) (Fig. 7d). This shows that powerful laser illumination can directly kill cells by destroying the cell envelope, and the species-related “destructive threshold” can serve as a boundary to separate the intracellular damage from the compromised structural integrity.

### Implications for Raman spectroscopy and other optical technologies.

**FIG 8:**
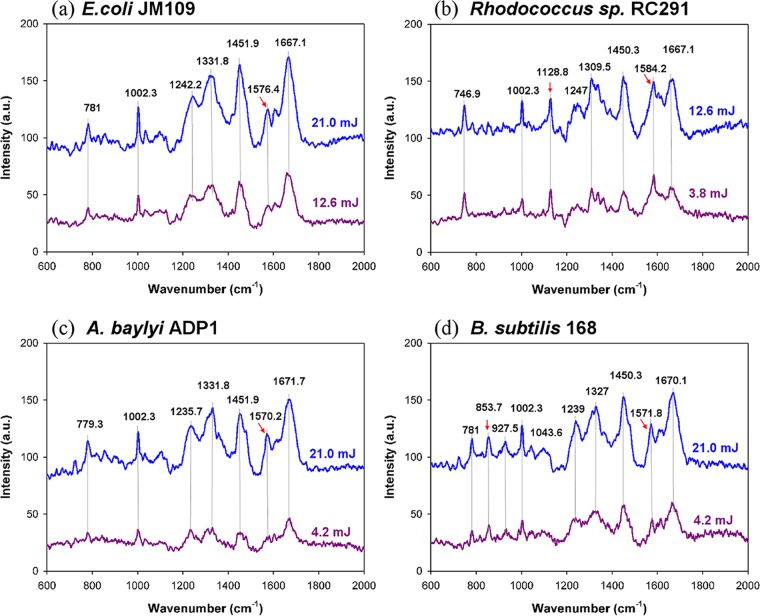
Raman spectra of E. coli JM109 (a), Rhodococcus sp. RC291 (b), A. baylyi ADP1 (c), and B. subtilis 168 (d) at two irradiation doses shown separately in blue and purple colors. These are averaged spectra from <10 cells. Red arrows indicate features whose positions differ significantly in the spectra of the four strains.

The results above illustrate that laser irradiation can jeopardize cell growth and viability, although this effect is dependent on the irradiation energy and cell type. This indicates caution should be exercised in the use of Raman spectroscopy and other optical technologies that involve the use of high laser energy. Here, initially, to evaluate whether meaningful single-cell Raman spectra can be achieved without fatal damage to cells, single-cell Raman spectra of the four strains were acquired using a relatively low irradiation energy, at which irradiated cells still showed growth on the basis of results as shown in Fig. 4. It was found that when a laser dose of ∼12 mJ was used for E. coli JM109, a high growth rate was maintained after irradiation (the possibly small reduction is not significant), whereas when using a much lower dose of ∼4.0 mJ for the other three strains, Rhodococcus sp. RC291, A. baylyi ADP1, and B. subtilis 168, the reductions in growth rate were much more significant, particularly in the case of A. baylyi ADP1 (Fig. 3b). In addition, it was found that to obtain “reference” single-cell Raman spectra with good signal-to-noise ratios, acquisition times around the “destruction threshold” had to be used for the three non-E. coli strains (Fig. 8). The assignments of the characteristic peaks were well documented previously (Fig. 8; see also Table S1) (22–24). Importantly, when using the lower energies that enabled cell growth after Raman measurement (i.e., 80% growth for E. coli JM109 and >40% growth for the others), these characteristic peaks were clearly present (Fig. 8, gray lines) with sufficient signal-to-noise ratios.

## DISCUSSION

Advanced optical imaging and manipulation technologies are used extensively in life science. Although it is often taken for granted that these technologies are noninvasive and do not impose detrimental side effects on living cells, there is increasing evidence indicating otherwise (18, 25, 26). In the field of Raman spectroscopy, Raman-activated cell sorting has emerged as a powerful label-free cell-sorting approach, linking single cells to their intrinsic phenotypic profiles and revealing cell functions (27). After cell sorting, it is often desirable to either cultivate or process cells for DNA sequencing. Since the intrinsic Raman signal is weak, it is crucial to find a trade-off condition which not only enables obtaining good-quality single-cell Raman spectra (SCRS) but also keeps cells alive after the Raman measurements.

Here, using simple microfluidic devices, we were able to quantitatively evaluate the effects of laser irradiation on bacterial cells at the single-cell level. This approach offers several unique advantages, including the application of focused irradiation of a known dose to each cell, real-time tracking of cell growth dynamics from the onset of irradiations, and a reliable comparison of a range of irradiation conditions in parallel. The capability of directly correlating irradiation with single-cell growth data provides invaluable insights into how a bacterial strain, i.e., a population, responds to a broad range of irradiation energies.

By evaluating both Gram-positive and Gram-negative species, we found a generic three-phase response of bacteria to laser irradiation: phase 1, the cells are inhibited but viable; phase 2, the cells are nonviable but intact; and phase 3, the cells showed physical dissolution. The quantitative single-cell growth study has enabled an estimation of the destructive thresholds where all the cells become nonviable but remain intact. Below the destructive thresholds, an increased irradiation dose results in a reduction in both the average cell growth rates and the strain culturability but an increase in the lag time. This phenomenon is similar to those observed when bacterial cells are under environmental stress, such as through exposure to antibiotics (16, 17, 19, 28). Indeed, phototoxicity due to the generation of singlet oxygen or reactive oxygen species is such a well-documented stress (13). As expected, continuously increasing the irradiation dose far beyond the destructive threshold inevitably led to the physical destruction of the cells regardless of whether the cells were from Gram-positive or Gram-negative species. This process appears to be associated with photothermal effects (29), since it was observed that the dissolution processes became faster with a higher laser dose.

Importantly, the dose range for viable cells in phase 1 and the destructive threshold are species dependent. In particular, the doses that Gram-positive strains can endure are much lower than those that the Gram-negative strains withstand, probably due to the different structures of their cell walls. For example, the cell walls of Gram-negative bacteria were reported to efficiently prevent the penetration of exogenous singlet oxygen generated from laser irradiation (30). Furthermore, Gram-negative and Gram-positive bacteria have different compositions of endogenous photosensitizers within their cells, such as light-absorbing porphyrins and flavins. These generate different levels of reactive oxygen species that can lead to fatal damage, such as the degradation of DNA bases (18, 30). For instance, E. coli exhibited exceptional photoinsensitivity and maintained ∼80% growth rates at ∼20 mJ, a value that is far beyond the destruction thresholds for the other strains. These findings highlight the significant variations between species as well as between individual cells within a population and thus justify a need for careful assessments for a given strain if its viability is of importance.

Although the Raman spectroscopic technique has been well accepted as a noninvasive method for microorganism detection (31), a recent study showed that cells that are directly impacted by the laser lose their membrane integrity (32). By directly correlating Raman spectra with single-cell growth characteristics, we demonstrated that strong Raman signals from nonpigmented bacterial cells are obtainable at doses that still allow continuous cell growth. This provides convincing evidence for the nondestructive characteristics of Raman spectroscopy on individual bacterial cells. However, moderate laser irradiation during Raman acquisition can exert an environmental stress on cells and can compromise cell growth in a dose-dependent manner. Considering the variations in the stress resistance of individual cells within a strain, a careful evaluation of Raman acquisition parameters (e.g., power and time) is needed for a given strain.

Furthermore, with *in situ* Raman detection of cellular uptake of deuterium (D) from a heavy water-containing medium, we showed the loss of metabolic activity of intact cells, revealing the early stage of destruction of cell viability by laser irradiation. This method overcame difficulties associated with the identification of dead cells with intact membranes which were present in the current study, i.e., cells in phase 2 as described above, and which were possibly present in many other studies (16, 17, 19). In contrast, the commonly used dead cell staining with propidium iodide depends on the penetration of the dye into cells, which often leads to inconsistent staining results (33, 34) and is likely to overestimate cell viability. However, heavy water is readily available and its addition to culture medium does not affect the growth of many bacterial strains (21). The integrated microfluidic and *in situ* Raman approach demonstrated here provides a simple sensitive determination of cell viability without the need of multistage fluorescence staining.

In conclusion, taking advantage of this single-cell microfluidic approach, we have quantitatively evaluated the effects of laser irradiation on individual bacterial cells and its implications in Raman spectroscopy. Laser irradiation can compromise the physiological function of bacterial cells, and the degree of destruction is dose dependent, ranging from reduced cell growth to the loss of metabolic activity and physical structural damage. On the basis of the quantitative single-cell growth data, the energy threshold for destruction is strain dependent, where Gram-positive bacterial cells are more susceptible than Gram-negative bacterial strains to their radiation-induced damage. Convincing evidence of nondestructive characteristics of Raman spectroscopy on individual bacterial cells was demonstrated. Although a Raman spectrum with a sufficient signal-to-noise ratio can be obtainable without causing cell death, the variety of responses from different strains and even from individual cells within the same population justifies a careful evaluation of Raman acquisition if the viability of a population is critical.

## MATERIALS AND METHODS

### Cell culture.

Single colonies of two Gram-negative species, Escherichia coli JM109 (Promega Co., UK) and Acinetobacter baylyi ADP1 (35), and two Gram-positive species, Bacillus subtilis 168 (36) and Rhodococcus sp. RC291 (37), were each cultured in LB (Luria-Bertani) broth (Sigma-Aldrich). E. coli JM109, A. baylyi ADP1, B. subtilis 168, and Rhodococcus sp. RC291 were grown at 37, 30, 37, and 28°C, respectively, overnight prior to microfluidic experiments.

### Microfluidic device fabrication.

Microfluidic devices were fabricated from polydimethylsiloxane (PDMS) elastomer as described previously (38). The devices consist of eight parallel shallow microchambers designed for trapping single cells and monolayer cell culture (Fig. 1). The chamber heights were tailored between 0.7 μm and 1.5 μm for different bacterial sizes. On one side of the chambers, barriers of ∼0.4-μm height were used to block cells in the chamber when loading the cells from the other side. On either side of the chambers, there was a channel (50 μm wide by 10 μm high) for delivering medium during cell culture.

### Monolayer culture of cells on chip.

Bacterial cultures were diluted with the same fresh medium and immediately loaded into the microchambers from one channel. After this, those left in both channels were flushed out with fresh medium at a speed of 5 μl/min. Thereafter, the medium flow rate was changed to 0.1 μl/min and maintained for 10 min prior to laser irradiation. The medium flow was stopped during laser irradiation and resumed afterwards. This continuous-flow culture provided constant nutrients to the cells and simultaneously eliminated metabolic waste. All on-chip cultures were performed at ∼26°C.

### Laser irradiation and Raman spectrum measurements.

A Raman spectrometer equipped with a Synapse charge-coupled-device (CCD) camera (LabRAM HR800; Horiba Ltd.) was used for both laser irradiation and Raman acquisition. After the laser beam became stable (>2.5 h with full power at ∼18 mW), it was focused on randomly selected single cells in the device through a 63× objective lens (numerical aperture [NA] = 0.7) for laser irradiation. The laser spot was experimentally determined to be 1.4 μm in diameter (i.e., 1.54 μm^2^) (data not shown). The irradiation energy (mJ = laser power [mW] × exposure time [s]) over this spot size is given in the text. The laser power was adjusted by changing the optical density of a filter located between the laser source and the microscope objective; a 1-s exposure time was used for the majority of experiments. In the case of irradiation levels higher than 18 mJ, the full power of the laser (∼18 mW measured at the objective) and increased exposure times were employed.

Single-cell Raman spectra were acquired using a 600 grating/mm and 1,000-μm pinhole. Raman spectra were directly acquired from cells cultured on a two-layered microwell microfluidic device (see sections below; see also Fig. S1 in the supplemental material). In this case, cells in the deep microwells were far enough from the PDMS wall to eliminate the interference presented by Raman signals from PDMS. However, for cells cultured in the shallow PDMS microchamber chips (Fig. 1), since PDMS gives strong Raman signals, the Raman spectra shown were not collected from cells in the on-chip device. Instead, spectra were collected from cells grown in the same batch culture as follows. Cells were washed with distilled water, centrifuged twice at 5,000 rpm for 5 min, and resuspended in water. Then, 2 μl of the cell solution was deposited on an aluminum-coated quartz surface and air dried for ∼15 min prior to Raman acquisition using exactly the same irradiation conditions (power and duration) as those used when irradiating the cells in microchambers.

### Calculation of specific growth rate and lag time.

Time-lapse imaging of cell growth in the microfluidic devices was conducted at ∼26°C for a period of ∼6 h. Monolayer culturing enabled us to determine the specific growth rate (μ [h^−1^]) of each single colony via the following equation as described previously (16, 17): μ = ln (*S_t_*/*S_0_*)/(*t* − λ), where *S_0_* and *S_t_* are the areas of a single colony at the initial time (*t* = 0) and at time *t*, and λ is the lag time of bacterial growth. To evaluate the percentage of cell survival after laser irradiation, cell culturability was calculated as the fraction of growing cells among the total illuminated cells within the experimental period.

### Statistics.

A total of 40 randomly selected colonies from at least three independent experiments were analyzed for each strain (note, each colony started from a single cell). Unless denoted, average values and standard deviations are given. All statistical analyses were performed using GraphPad Prism software. An unpaired one-way analysis of variance (ANOVA) was used for each species. A difference was regarded as significant when the *P* value was <0.05.

## Supplementary Material

Supplemental material
