# Supplementary material for: Effect of Laser Irradiation on Cell Function and Its Implications in Raman Spectroscopy
Source: Appl Environ Microbiol. 2018 Apr 2;84(8):e02508-17. doi: 10.1128/AEM.02508-17 (PMC5881070; doi:10.1128/AEM.02508-17)
Supplement: Supplemental material [file supp_84_8_e02508-17__index.html]

Effect of Laser Irradiation on Cell Function and Its Implications in Raman Spectroscopy — Supplemental material 

# Effect of Laser Irradiation on Cell Function and Its Implications in Raman Spectroscopy

## Supplemental material

- Supplemental file 1 -

  Protocols; structure of the microwell microfluidic device (Fig. S1); assignments of the observed Raman bands in Fig. 8 (Table S1).

  PDF, 382K
