# Supplementary material for: Effect of Laser Irradiation on Cell Function and Its Implications in Raman Spectroscopy
Source: Appl Environ Microbiol. 2018 Apr 2;84(8):e02508-17. doi: 10.1128/AEM.02508-17 (PMC5881070; doi:10.1128/AEM.02508-17)
Supplement: Supplemental material [file AEM.02508-17_zam008188433s1.pdf]

# Supplementary material

## Effect of laser irradiation on cell function and its implications in Raman spectroscopy

Xiaofei Yuan[a], Yanqing Song[a], Yizhi Song[b], Jiabao Xu[b], Yinhu Wu[b], Andrew Glidle[a], Maggie Cusack[c], Umer Z. Ijaz[a], Jonathan M. Cooper[a], Wei E. Huang[b] and Huabing Yin[a*]

*School of Engineering, University of Glasgow, Glasgow G12 8QQ, UK [a]; Department of Engineering Science, University of Oxford, Parks Road, Oxford OX1 3PJ, UK [b]; Division of Biological & Environmental Sciences, Faculty of Natural Sciences, University of Stirling, Stirling, FK9 4LA, UK [c]*

*Corresponding author: huabing.yin@glasgow.ac.uk; Telephone: +44 (0)1413304109

This file contains protocols for live/dead assays, fabrication of the microwell microfluidic devices, and *in situ* monitoring cell metabolic activity using Raman spectroscopy. It also includes the assignments of the observed Raman bands in Figure 8.

## TABLE OF CONTENTS:


| | |
|---|---|
| Protocol: Live/dead staining | S-3 |
| Protocol: Fabrication of the microwell microfluidic devices | S-3 |
| Protocol: *In situ* monitoring cell metabolic activity using Raman spectroscopy | S-3 |
| Figure S1: Structure of the microwell microfluidic device. | S-4 |
| Table S-1: Assignments of the observed Raman bands in Figure 8. | S-4 |
| References | S-4 |

**PROTOCOLS**

***Live/dead cell staining.*** Propidium iodide (1 mM in DMSO, Invitrogen) and SYTO 9 green fluorescent nucleic acid stain (5 mM in DMSO, Invitrogen) were used for live/dead staining after cells were exposed to laser and then cultured on chip. At the end of culture, LB medium was changed to the staining solution (2 μl of propidium iodide and SYTO 9 mixture each in 1 ml of LB medium) and the flow rate was maintained at 0.1 μl/min. The staining process lasted for ~ 1.5 hours. SYTO9 (green) was detected using 470-795 nm (excitation)/ 510-550 nm (emission) and propidium iodide (red) using 503-552 nm (excitation) /607-695 nm (emission) respectively.

***In situ monitoring cell metabolic activity using Raman spectroscopy.*** To evaluate metabolic activity of non-growing cells, after laser irradiation under the conditions that stop cell growth, normal LB medium was changed to a LB medium containing 90 % heavy water ($D_2O$, 99.9 atom % D, Sigma-Aldrich) before the post culture for 6 hours. A double-layered microfluidic device was used in this case, which consists of deep microwells (~250 μm deep, 0.5 mm in diameter) within a high channel (100 μm high) and a quartz substrate (see below, Fig. S1). This was to avoid strong Raman background from PDMS and glass as seen in the shallow chambered microfluidic device (Fig. S1). Before Raman acquisition, the $D_2O$-medium was removed using a PBS buffer flow. A small pinhole (100 μm) and a long acquisition time (120 s) were used for Raman acquisition to ensure sufficient signal collection.

**Fabrication of microwell-structured microfluidic devices.**
Polydimethylsiloxane (PDMS)/curing agent (*Sylgard* 184; Dow Corning Co., U.K.) (10/1) mixture was spin-coated on a silanized silicon wafer at 380 rpm × 30 s to make a thin PDMS film (~ 250 μm thick). After curing at 120 ºC for 10 min, it was peeled off and then holes of 0.5mm in diameter were punched in it using a biopsy punch. Thereafter it was cleaned and bonded to a clean quartz slide after oxygen plasma treatment at 100 W for 30 s. In parallel, the same PDMS/curing agent mixture was poured into a silanized SU8 mould consisting of a microchannel of 100 μm high. After curing at 80 ºC for 2h, the PDMS replica was peeled off from the mould, holes were punched for both inlets and outlets, and it was then cleaned and bonded to the quartz slide with the thin well-PDMS film on it. The microchannel was on the top of microwell areas as shown in Fig. S1.

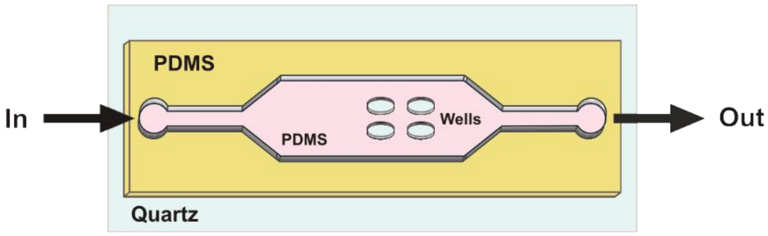


**Figure S1.** Schematic illustration of a microwell-structured microfluidic platform.

**Table S-1.** Observed Raman bands of four types of species and their tentative assignment. (1-3)

| Raman shift ($cm^{-1}$) | Band assignment |
|---|---|
| 1667-1671 | Amide 1 (mainly C=O str) |
| 1584 | Cytochrome c |
| 1570-1576 | Guanine, adenine (ring str) |
| 1450-1452 | $CH_2$/$CH_3$ (def) |
| 1332 | Guanine (ring str) |
| 1327 | $CH_2$ (wag) |
| 1310 | Cytochrome c; $CH_2$/$CH_3$ (tw, wag, bend of lipids) |
| 1235-1247 | Amide III (N-H in plane bend and C-N str) |
| 1128 | Tyrosine, phenylalanine |
| 1040-1100 | Carbohydrates |
| 1002 | Phenylalanine |
| 853 | Tyrosine |
| 779-781 | Cytosine, urasil (ring, str) |

**References:**

1. De Gelder J, De Gussem K, Vandenabeele P, Moens L. 2007. Reference database of Raman spectra of biological molecules. J Raman Spectros 38:1133-1147.
2. Huang WE, Griffiths RI, Thompson IP, Bailey MJ, Whiteley AS. 2004. Raman Microscopic Analysis of Single Microbial Cells. Anal Chem 76:4452-4458.
3. Maquelin K, Kirschner C, L.P. C-S, van den Braak N, Endtz HP, Naumann D, Puppels GJ. 2002. Identification of medically relevant microorganisms by vibrational spectroscopy. J Microbiol Methods 51:255-71.
